# The effect of picture book reading on young children’s use of an emotion regulation strategy

**DOI:** 10.1371/journal.pone.0289403

**Published:** 2023-08-02

**Authors:** Johanna Schoppmann, Franziska Severin, Silvia Schneider, Sabine Seehagen

**Affiliations:** 1 Department of Developmental Psychology, Faculty of Psychology, Ruhr University Bochum, Bochum, Germany; 2 Department of Clinical Child and Adolescent Psychology, Faculty of Psychology, Ruhr University Bochum, Bochum, Germany; University of Amsterdam: Universiteit van Amsterdam, NETHERLANDS

## Abstract

Picture book reading is an enjoyable everyday activity for many young children with well-known benefits for language development. The present study investigated whether picture book reading can support young children’s social-emotional development by providing a learning opportunity for the usage of emotion regulation strategies. Three-year-old children participated in two waiting situations designed to elicit negative affect. Between these waiting situations they read a picture book. In two experimental conditions, the book depicted how a protagonist (same-aged peer or young adult, respectively) waited for a desired object and distracted herself with toys while waiting. Children in an additional control condition read a picture book that was unrelated to waiting. Use of distraction did not differ between conditions. Parents often read picture book interactively with their children. Therefore, in an additional condition (Exp. 2), the experimenter read the picture book featuring the same-aged peer protagonist in an interactive way intended to facilitate transfer. Apart from the reading style, the design was identical to experiment 1. Experiment 2 intended to test whether changes in reading style lead to differences in three-year old children’s social-emotional learning from picture books. When controlling for the children’s picture book experience, children in the experimental conditions exhibited an increase in distraction in contrast to children in the control condition. In sum, results suggest that picture book reading could be an ecologically valid and versatile method for supporting 3-year-old children in their use of an age-appropriate adaptive emotion regulation strategies such as distraction.

## Introduction

Joint picture book reading is not only an enjoyable everyday activity for many young children but provides different types of learning opportunities [1]. Toddlers and young children are able to acquire some information from picture books and apply this information to the real world. For example, 18-, 24- and 30-month-old children imitated a multi-step action sequence 24 hours after they saw a protagonist in a picture book demonstrate these target actions, with an age-related increase in imitation scores [2]. In another study, 4-year-old children learned information about animals from a book and applied the information to living animals [3]. In this study, the experimenter and children read a picture book about a predatory bird which could not detect its prey, as the prey was camouflaged at first. But the bird found its prey later when it saw the prey sitting in front of a differently colored background. Children subsequently chose a real display tank for a lizard that matched its color in order to protect it from the picture of the hungry bird [3].

The literature also suggests that not all reading situations provide equal learning opportunities. Variations in interaction styles and pictures can have a significant impact on the reading experience and resulting learning. For example, dialogic reading can be conceptualized as asking children questions, helping them extract themes or prompting them to explain [4, 5]. When children got older, parents tended to talk more about the pictures and stories in the picture book, and to ask further questions about these [1, 6, 7], supporting a better understanding and facilitating language acquisition. Within the same age-group, an interactive reading style was more effective in promoting language development than pure text reading [8, 9]. In a recent review on factors that influence children’s ability for information transfer from books to real life, the authors concluded that the most supportive element was to have conversations about the books while reading [4]. Furthermore, understanding and transfer of information were easier for children when pictures in the book were similar to the corresponding objects in the real world in contrast to black and white graphics, cartoons, drawings, or pictures with additional manipulative features, such as “pop-ups” [10–12]. In sum, there is ample evidence that young children learn and remember facts, actions and vocabulary from picture books. Both features of the book itself and of the interactions around the book can help or hinder learning.

These findings can be regarded as the base for an emerging line of research that considers whether children take information from picture books and adapt their *social* behavior. One such study assessed whether classic moral stories can promote honesty in children [13]. Four different picture books were read to 3- to 7-year-old children, followed by a task were children had an opportunity to lie or tell the truth. Only one of the classic stories promoted truth telling. The effective story focused on the positive consequences of truth telling while the other stories focused on the negative consequences of lying. This study suggests that young children are, in principle, able to transfer moral knowledge from picture books to adapt their own behavior. A second study found that 4.5– to 5.5-year-old children learned novel words, moral lessons, and story details from shared picture book reading with their parents [14]. In this study, children were asked about the moral lesson, but it was not behaviorally tested whether they applied it. A third study investigated whether picture book reading affected 4- to 6-year-old children’s prosocial behavior [15]. Here, children acted more altruistically after reading a picture book with human in contrast to anthropomorphized animal characters. A further study found that preschoolers improved in their expression of emotion terms and their ability to connect thoughts to emotions, to define social problems and to generate solutions after having taken part in five meetings in which they discussed the social and emotional conceptualizations of a picture book in contrast to children in a control group who discussed the book’s plot, characters and actions [16]. These studies demonstrate that the benefits of picture book reading not only concern cognitive functioning but can also promote social behavior. Children’s social and emotional behaviors and experiences are fundamental aspects of development, and emotion regulation is considered to be essential for both domains [17, 18]. To the best of our knowledge, there have been no attempts yet to specifically teach young children emotion regulation strategies via picture book reading.

Emotion regulation can be defined as `the process of modulating the occurrence, duration, and intensity of internal states of feeling (both positive and negative) and emotion-related physiological processes`[19, p.1]. Its development is a complex process that starts in infancy and continues throughout the lifespan [20, 21]. Successful emotion regulation is fundamental for life in its entirety [22, 23], including but not limited to adaptive functioning [24] and in mastering everyday-challenges such as summoning the courage to attend a job interview [25]. Links to the development of mental disorders also exist [e.g., 20, 26, 27].

According to the tripartite model of family influences on emotion regulation, children learn emotion regulation mainly through three pathways, namely observation, emotion-related parenting practices and the emotional climate of the family [19, 28]. Moreover, according to the model, parent characteristics and child characteristics have an impact on these pathways and on child emotion regulation and adjustment. In the present study, we focused on one of these routes–observation—in the context of picture book reading. In the framework of the tripartite model, observation includes modeling, social referencing, and emotion contagion [28]. Young children often learn new behaviors by copying others around them, as it eliminates the need for lengthy trial and error learning [29]. According to Bandura’s prominent social learning theory, young children learn most behaviors through imitation [30]. Recent research with 2-year-olds has shown that toddlers are able to apply the emotion regulation strategy distraction when being mildly frustrated after having observed an adult model do the same [31, 32]. Distraction is an emotion regulation strategy that is widely considered to be helpful for young children in frustrating situations [33–35] and was defined as an interaction with a different object or talking about something else than the negative emotion eliciting stimulus. Its use relates to better compliance and less negative affect [36, 37].

The present study tested if 3-year-old children would alter their use of a particular emotion regulation strategy, namely distraction, as a result of reading a picture book that illustrates its use. All children participated in two waiting situations, which reliably induce negative affect [31, 32, 34, 38]. Their emotion regulation strategies were assessed in each of these situations. Between these two waiting situations, children in the experimental conditions participated in a book reading interaction, depicting either a child or an adult model who distracted themselves while having to wait. Children in an additional age-matched control condition read a book unrelated to the waiting situations. We predicted that children in the experimental conditions, but not in the control condition, would show increased use of distraction during the second waiting situation. We had no specific predictions as to whether the child or the adult model would be more effective in eliciting increased use of distraction. While the analyses regarding observational learning were more confirmatory [cf., 31, 32], they also had an exploratory aspect, as learning of an emotion regulation strategy from a picture book had not been assessed in this age-range before. Analyses regarding the age of the protagonist were rather exploratory. In a follow-up experiment (Exp. 2), we included a further condition in which the girl protagonist picture book was read in a dialogic manner in order to investigate whether differences in reading style would influence subsequent child behavior.

## Experiment 1

### Method

#### Participants

Overall, *N* = 80 children participated. Of these, *n* = 8 were excluded due to experimenter error, *n* = 3 due to an interruption of the waiting situation, when children needed to use the bathroom, *n* = 2 due to children managing to get snacks out of their parent’s bag and eating them during the waiting situation, and *n* = 1 child due to not wanting the desired object. Sample size was determined a priori using G* power [39], assuming a medium-sized interaction effect *f* = .25, power = .95, correlations among repeated measures = .5, *p* = .05 in the main ANOVA, resulting in a total of *N* = 66 children (*n* = 22 in each condition). The final sample therefore consisted of *N* = 66 (*n* = 33 boys) 36-month-old (+/- 1 month) children. Of these, *n* = 57 children took part with their mothers and *n* = 9 children took part with their fathers. Mother’s mean age was *m* = 35.52 years (*SD* = 4.29) and father’s mean age was *m* = 38.83 (*SD* = 5.54). Sixty-eight percent of mothers and 58% of fathers had a university degree. Ninety-four percent of mothers and 91% of fathers spoke German as their native language. We did not collect data on ethnicity. Sixty percent of children had one or more siblings.

Participants were recruited from a database containing contact details of caregivers who had expressed an interest in participating in developmental studies with their child. Specifically, all families whose child was born in a specific timeframe living in the city of Bochum, Germany received a letter explaining the University’s research program on child development and were invited to participate with their child. In the present study, parents received a 5 € reimbursement and children received the snacks and gift from the waiting situations, further coloring pages as a present and a certificate for their participation. Study procedures were approved by the local Ethics Committee of the Ruhr University Bochum. Written parental consent was provided before participation. Data collection took place between 2017 (May) and 2018 (May). Anonymized data can be found at OSF (https://osf.io/f8zrt/).

#### Design

**Fig 1:**
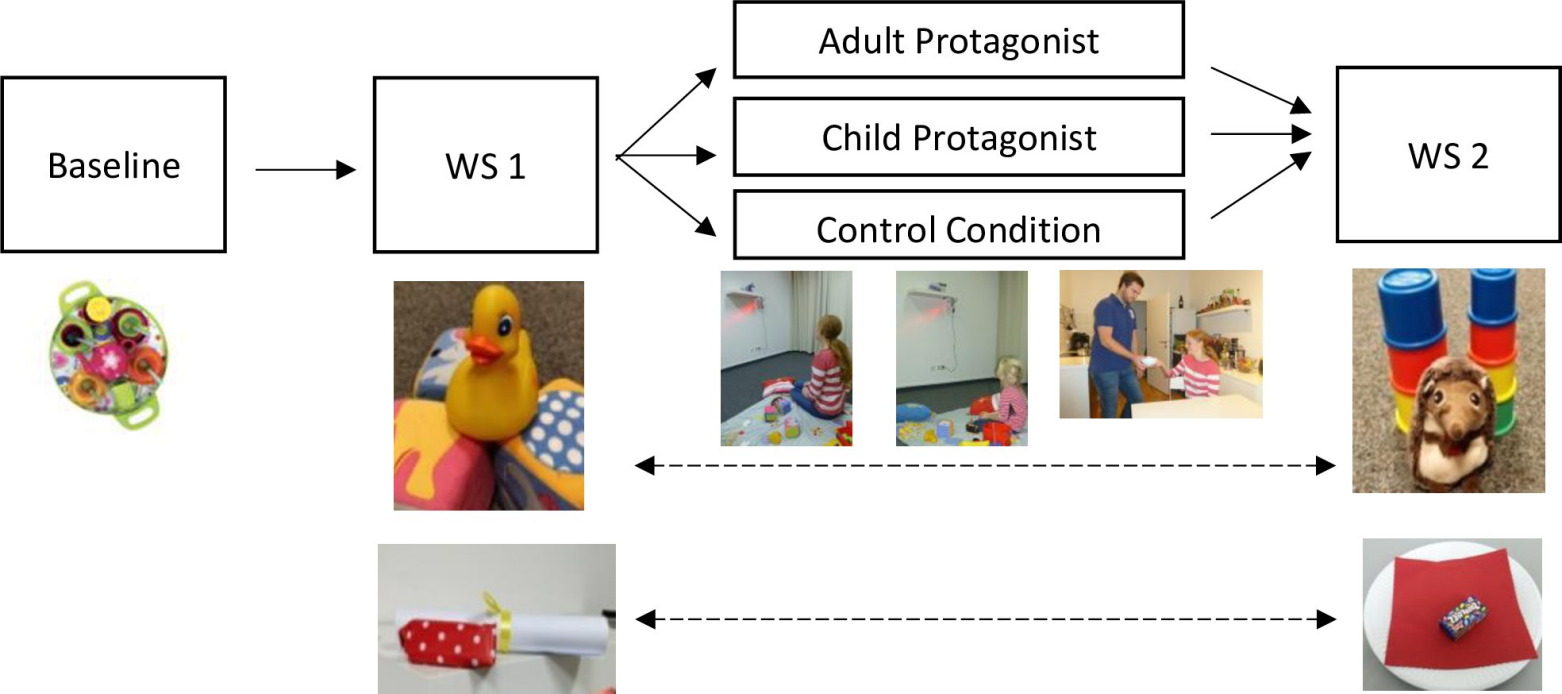
Study design and general procedure. Order of both toy sets and waiting stimuli in waiting situation (WS) 1 and waiting situation (WS) 2 was counterbalanced.

Children were randomly assigned to one of three conditions (two experimental conditions: adult protagonist, child protagonist; one control condition). After warm-up, all children participated in a baseline free-play situation with their accompanying parent. Afterwards, each child participated in two waiting situations (snack delay, gift delay; order randomized). Hence, the design included one between-subject factor (condition) and one within-subject factor (waiting situation). Parents completed questionnaires but remained in the same room with their child throughout the procedure. In between the waiting situations the experimenter read a picture book with the children. Its content varied between conditions. Fig 1 displays the design and overall procedure.

#### Materials

*Desirable objects.* The parent chose one snack the child liked out of five options: smarties, gummy bears, raisins, cookies or pretzels. The snack was presented on a plastic plate with a red napkin. The gift consisted of three crayons and a coloring page. The crayons were wrapped in colorful wrapping paper and the coloring page was rolled up with a yellow bow attached to it.

*Toys*. In the baseline situation, the parent and child played with a toy picnic set. There were two further sets of toys for the waiting situations. Their order was randomized (Fig 1). Toys were chosen with the aim of not being too interesting, as the waiting situations were intended to elicit negative affect. At the same time, however, children needed to have an opportunity to distract themselves.

*Picture books.* There were four different picture books, produced specifically for the present study. Each book consisted of six laminated DIN-A4 pages, each page containing a 10 x 15 cm photograph and 2–3 sentences [cf., 12].

**Fig 2:**
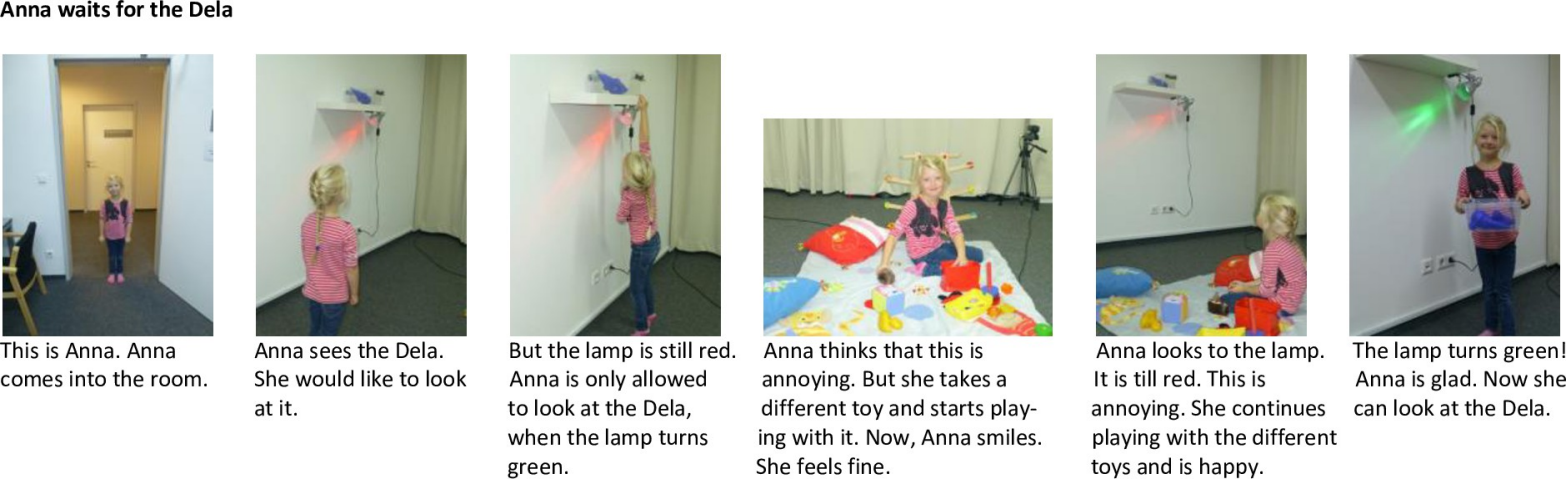
Picture book experimental condition, girl protagonist. This figure depicts the pictures and text of the pages of the picture book in the experimental condition with the girl protagonist.

**Fig 3:**
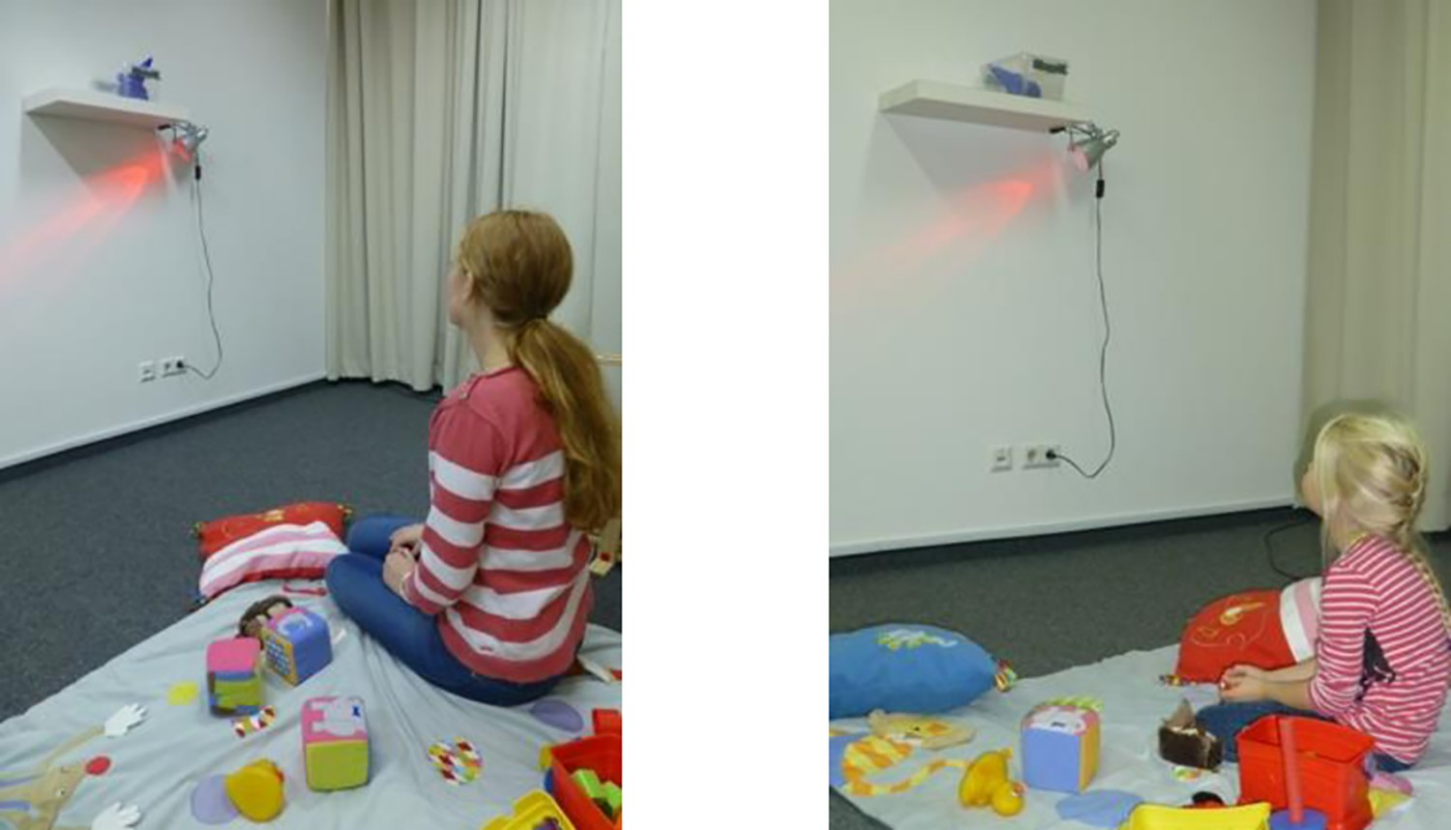
Example of adult protagonist picture book. Example of one page of the picture book (p. 5) in both experimental conditions. Pictures of protagonists were matched.

***Experimental conditions.*** The story in two of the picture books (experimental conditions) was set in the same laboratory in which the study took place. The storyline in the picture book was very similar to the events that occurred during the waiting situations in which the children participated. Specifically, the protagonist distracted herself with toys while waiting (Fig 2). Both picture books were highly similar to each other, except that the protagonist was a female adult in one condition and a five-year old girl in the other condition (Fig 3).

**Fig 4:**
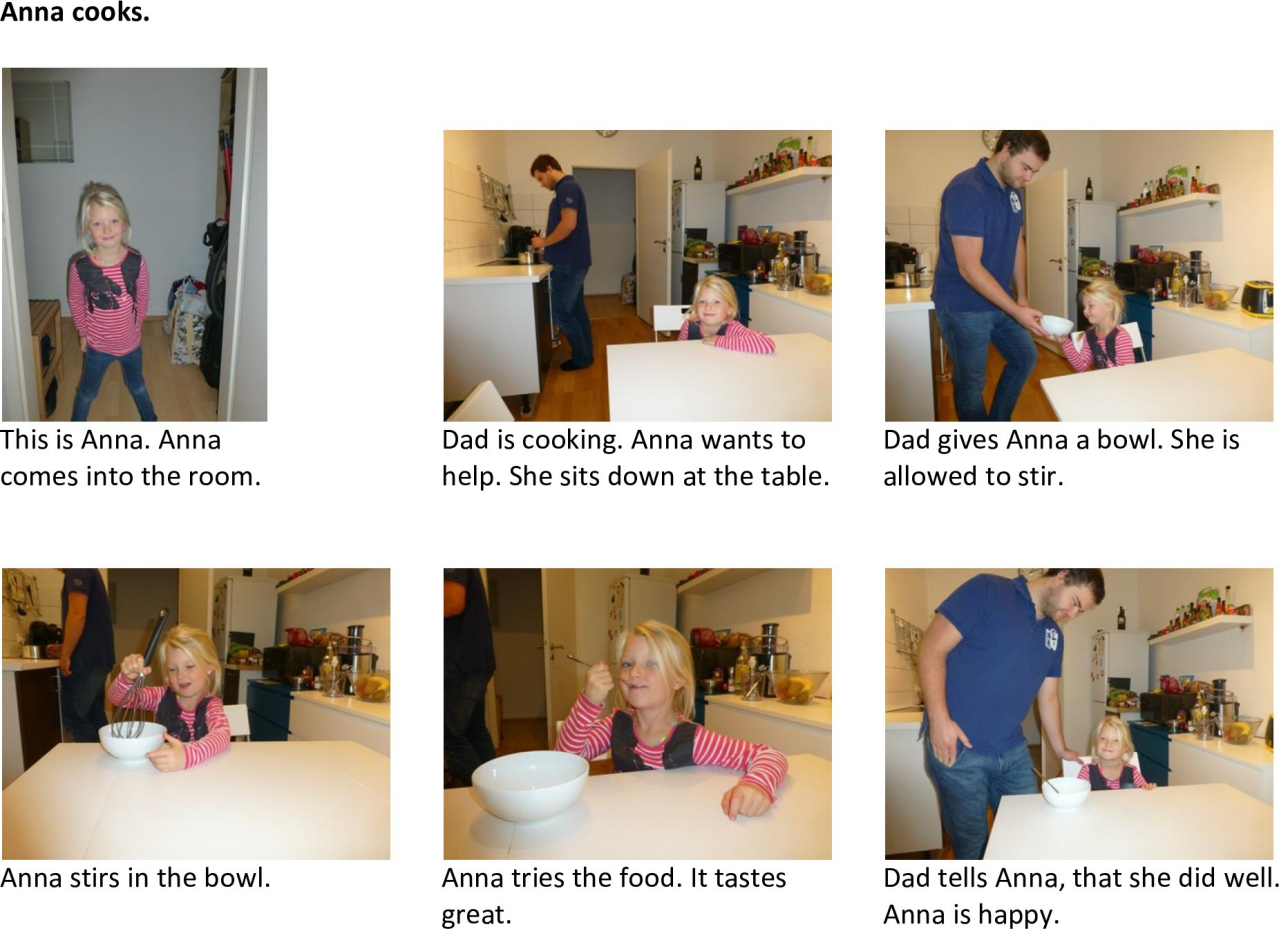
Picture book of the control condition. Pictures and text of the picture book in the control condition with the girl protagonist.

**Fig 5:**
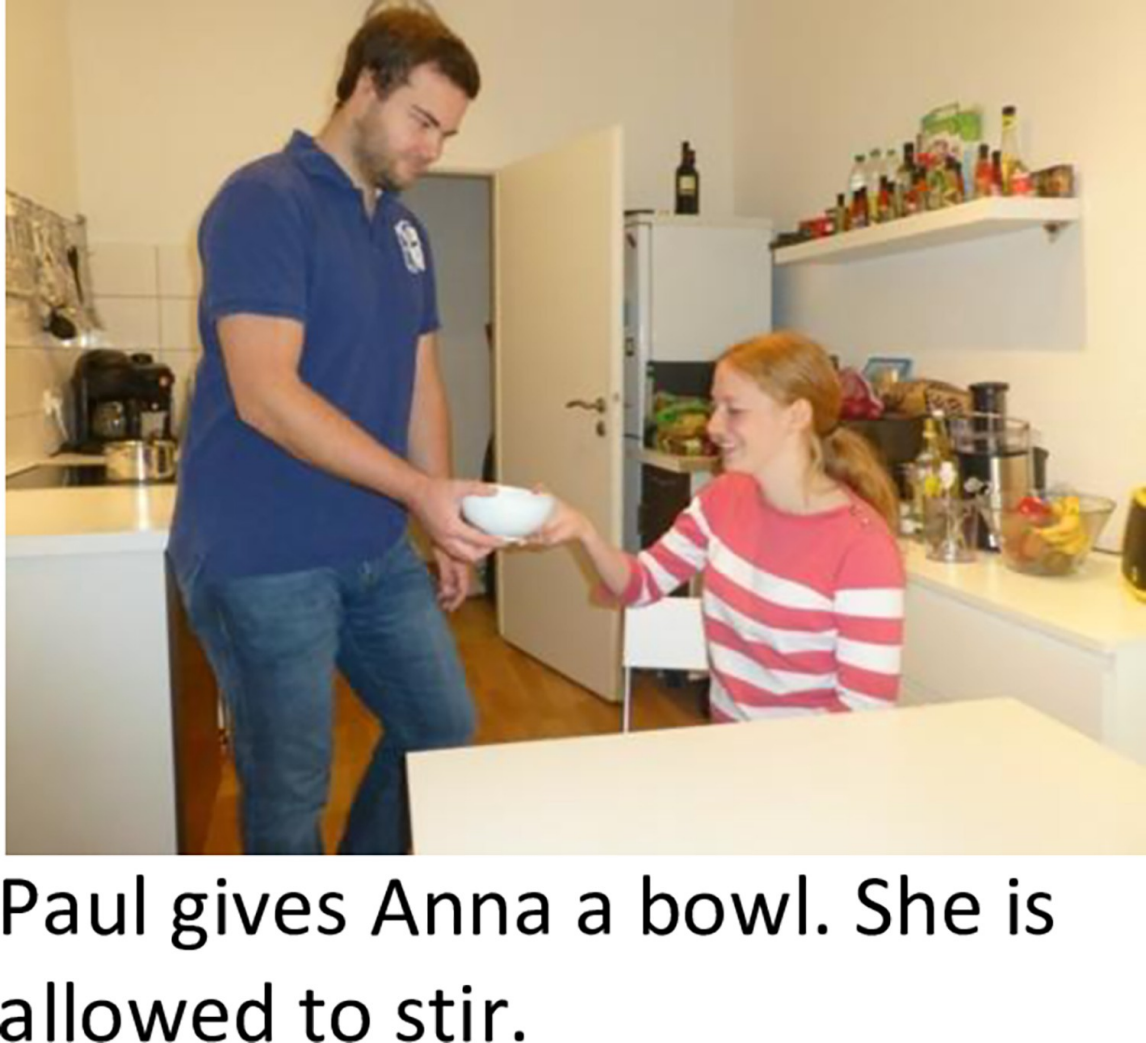
Sample picture of the adult protagonist book for the control condition.

***Control condition*.** In the control condition, there were also two similar picture books which were identical except for the identity of the protagonist (i.e., female adult, girl). The child and adult protagonists were the same as in the experimental conditions and picture books also had the equivalent length. The story was set in a kitchen where the protagonist helped a male adult to cook (Figs 4 and 5).

*Questionnaires*. ***Mental state assessment.*** Each child’s mental state was assessed by parent ratings immediately after baseline and both waiting situations using an adapted version of the ‘Multidimensionaler Befindlichkeitsfragebogen’ (MDBF). The MDBF is a questionnaire containing the scales ‘Good/Bad Mood’, ‘Calm-Agitated’ and ‘Wakefulness/Tiredness’ [40]. Only the scales ‘good/bad mood’ and ‘calm-agitated’ were used in the present study (example for the scale Calm-Agitated: “While my child was waiting for the snack, he/she felt relaxed”). Each scale consists of eight items and yields scores varying between 8 and 40 points in total. A high score indicates good mood and calmness, respectively. Re-test reliability ranges from *r* = .69 to *r* = .86 and internal consistency from *α* = .73 to *α* = .89 [40]. In the present sample internal consistency was *α* = .89 for ‘Good/Bad Mood’ and *α* = .89 for ‘Calm-agitated’.

***Picture book experience questionnaire.*** Parents were asked to rate four questions about the child’s experience with picture books. The items were: 1) How many picture books does your child own? (possible answers: <5, 5–10, 10–15, 15–20, >20). 2) How much time does your child spend reading picture books on an average day (possible answers: not at all, <30 minutes, 30–60 minutes, 1–2 hours, >2 hours). 3) How well does your child understand a new picture book? (possible answers: very well, well, average, poorly, not at all). 4) How much does your child like to read picture books? (possible answers: not at all, not very, neutral, a little, a lot).

#### Set-up and procedure

Altogether, there were four different female experimenters who were trained to conduct the study, though each child met with only one experimenter. Every time the experimenter left the room, she explained it to the child. When the experimenter cleared the room and went to bring new toys or the desired stimuli of the waiting situations, she said: ‘I have something else for you, I will be back soon.’

*Set-up.* In the laboratory, a play mat with two cushions and toys for warm-up were placed in the middle of the room. Moreover, a table with one chair was arranged in a corner of the room. There were four cameras partly hidden behind curtains. Furthermore, there was a wall-mounted shelf out of reach for the children (1.40m height) to which a lamp was attached. The lamp could be switched from red to green via remote.

*Warm-up.* Upon arrival the experimenter greeted the parent-child dyad and accompanied them to the laboratory. During warm-up, the experimenter played with the child and informed the parent about the study procedures both verbally and in writing. The parent received the opportunity to ask questions, provided written consent and chose the child’s favorite snack.

*Baseline situations and instructions*. During the 4-minutes baseline situation, the parent-child dyad played with picnic toys. The experimenter left the room and monitored the situation from an adjacent room on a screen. After the baseline situation, the experimenter returned, instructed the parent about their required behavior during the waiting situations and asked them to fill out questionnaires at the table. The parent was instructed to remain passive and, if addressed by the child, tell the child that they had no time as they had to fill out questionnaires. If the child performed forbidden activities, such as trying to touch the cameras or power sockets, the parent was to intervene and afterwards return to the questionnaires. After instructions, the experimenter continued playing with the child with the picnic toys until she felt certain that the child was comfortable. This moment usually constituted of the child actively engaging (that is, playing and talking) with the experimenter, while the parent sat at the table in the corner of the room. If the experimenter was unsure whether the child had settled in, she asked the parent and either continued playing to further settle in the child or continued with the procedure, depending on the parent’s reply. She then asked the child to describe what they could see on three different colorful pictures. Respective answers were not relevant for the present study and will not be considered further.

*Waiting situation 1*. Procedures for the waiting situations were identical to those conducted and described by Schoppmann et al. [31]. The experimenter cleared the room of all toys and returned with toy set 1 or 2, respectively. She played with the child for two minutes and then brought the snack or gift, depending on randomization. If it was the gift, she showed it to the child and explained that this was a gift for him or her. When the child reached for the gift, the experimenter quickly pulled it away and put it on the shelf. If it was the snack, the child was allowed to eat one item. When the child reached for the second item, the experimenter pulled the snack away and laid it on the shelf–out of the child’s reach. She then told the child that she had forgotten something outside and was going to retrieve it, and that the gift/snack on the shelf was his or hers, respectively. The child was going to receive it on the experimenter’s return. The experimenter was to return when the red lamp turned green. She then left the room, locked the door and monitored the situation from an adjacent room on a screen. After three minutes, the experimenter returned, inconspicuously switched the red lamp to green and the child received the gift or snack, respectively.

*Picture book reading.* The room was cleared of all toys and the experimenter returned with the picture book. The child and the experimenter read the book on the play mat facing the shelf with the lamp switched to red again. The experimenter read the text in the book and replied to a child’s possible comments with a friendly but neutral `hmm`. The picture book was read twice in succession. Afterwards the experimenter asked control questions. In the experimental conditions, she asked: ‘What is Anna waiting for?’ and ‘What does Anna do while waiting?’. In the control condition, the experimenter asked: ‘Who is cooking?’ and ‘What does Anna do?’. Both questions were asked a second time if the child did not answer or gave a wrong answer the first time. If there was no correct answer after the second repetition, the experimenter opened the relevant page in the picture book and stated the correct answer, thereby ensuring that all children understood the main message.

*Waiting situation 2.* The second waiting situation was very similar to the first waiting situation, except that the gift or snack and the toy set had not been used in the first waiting situation.

#### Data coding and interrater reliability

The mental state questionnaire was scored according to author instructions, calculating sum scores. The procedure was recorded by video cameras and coded using the software Interact which allows frame-by-frame analyses [41] (Mangold International, 1998). The last three minutes of the Baseline situation were analyzed to measure negative affect. Both waiting situations were coded for both negative affect and distraction.

*Coding of the control questions.* The replies to the control questions after the picture book readings were scored according to the following scoring scheme: Children received a score of ‘1’ for the correct answer after the question was asked for the first time, a ‘2’ after it was asked for the second time, a ‘3’ if they stated the answer while seeing the relevant page in the picture book and a ‘4’ when the experimenter had to give the correct answer.

*Coding schemes and interrater reliability.* Coding schemes were based on prior work [31]. A detailed coding scheme can be obtained from the first author upon request. For both constructs, their duration was measured. If measures overlapped e.g., a child whined and performed forbidden behaviors at the same time, overlaps were calculated using the software Interact and subtracted from the overall score. The final score therefore demonstrated the duration of the construct in each 3-minutes episode. All calculations were based on these scores.

All videos were coded by the first author. Twenty-eight videos (42%) were also coded by two different independent raters. Interrater reliability for distraction was ICC (one way random) = .98 and for negative affect ICC (one way random) = .81.

***Distraction*** Distraction was defined as consisting of both verbal and behavioral distraction. The coding scheme was based on a coding scheme used in a younger sample [31] but had to be expanded to factor in the children’s larger vocabulary skills. Verbal distraction was now coded as talking about objects, the picture book (only in the second waiting situation), role playing games, verbalizations of activities (i.e., `I am jumping`, `I am building a tower`), talking about experiences outside the laboratory (such as talking about other children in the kindergarten), singing using identifiable words (humming or singing using fantasy words was not coded). Directly talking to the parent or calling the parent was not coded as distraction. State verbalizations such as ‘I am tired’ or ‘I do not want to wait’ were also not coded as distraction. Behavioral distraction was coded as an interaction with an object during which the child had to simultaneously look at the object, touch the object with any body part and move the object or the adjacent body parts, such as moving the duck with the feet, while looking at it. Parental interactions and focus on the waiting stimuli such as the lamp or the gift were not coded as either verbal or behavioral distraction. The literature has distinguished between several emotion regulation strategies, whereby parent orientation and focus on the stimuli were not categorized as distraction strategies [42].

***Negative affect.*** Negative affect was coded either as verbal, when the child screamed, cried, whined, or through actions, when the child tried to leave the room resolutely or performed previously forbidden activities by their parents, such as touching electric sockets, switching off the lights or hiding behind the curtains. In terms of negative affect, attempts to leave the room included banging (but not gently knocking) against the door or hanging on the door handle. The coding scheme was taken from prior research [31].

### Results and discussion

#### Manipulation check and preliminary analyses

In order to ascertain that the waiting situation had successfully induced negative affect, we performed manipulation checks using both parental ratings and behavioral observations. Moreover, as an inclusion criterion, all children had to look at the stimulus when it was placed on the shelf.

First, we performed a paired t-test to compare duration of negative affect (in seconds) during the baseline situation and the first waiting situation which revealed a significant increase of negative affect (*M*_*Baseline*_ = .22, *SD*_*Baseline*_ = 1.12; *M*_*WS1*_ = 3.43, *SD*_*WS1*_ = 6.79), *t*(65) = -3.97, *p* < .001, *d* = .48. Second, we performed paired t-tests for the mental state assessment which showed increases in agitation and bad mood as rated by the accompanying parent, *t*(62) = 6.51, *p* < .001, *d* = .82 for agitation and *t*(62) = 5.38, *p* < .001, *d* = .68 for bad mood. Descriptive data for each condition can be found in Table 1. Further analyses regarding negative affect during different phases of the procedure can be found in the S1 File.

**Table 1:** Duration of negative affect.

Condition	*Negative affect (baseline)*	*Negative affect* (WS1)	*Negative affect* (WS2)	*MDBF calmness (baseline)*	*MDBF calmness (WS1)*	*MDBF calmness (WS2)*	*MDBF mood (baseline)*	*MDBF mood (WS1)*	*MDBF mood (WS2)*
Adult Protagonist (*n* = 22)	.42 (.20)	2.97 (6.84)	5.10 (13.42)	34.55 (5.03)	27.86 (8.00)	26.68 (7.91)	37.14 (3.67)	33.09 (6.13)	33.41 (4.84)
Girl Protagonist (*n* = 22)	.62 (1.89)	2.87 (5.41)	3.84 (8.85)	34.05 (4.99)	28.14 (7.51)	27.45 (8.06)	37.67 (2.50)	33.45 (5.55)	33.05 (7.81)
Control (*n* = 22)	0 (0)	4.45 (4.39)	4.39 (9.06)	34.48 (5.22)	27.14 (6.81)	25.73 (6.08)	37.76 (1.87)	33.24 (5.09)	33.09 (4.76)

Means and standard deviations (in brackets) of negative affect (in seconds) and parental ratings in baseline and both waiting situations (WS1; WS2).

Third, we calculated independent t-tests assessing possible differences in distraction and performance in answering the control questions between the children who read the picture book with a girl protagonist and those who read the picture book with a woman protagonist in the control condition. There were no differences, largest *t(20)* = 1.33, *p* = .199.

Fourth, we performed Kruskal-Wallis tests across conditions to check whether there was a difference in replies to the two control questions after having read the picture book. The distribution of replies to the control question can be found in Table 2. For question 1, there was a significant difference between conditions, χ2 = 15.54, *p* < .001, *η*_*p*_^*2*^ = .253 and the same was the case for the second question, χ2 = 10.31, *p* = .006. Dunn-Bonferroni post-hoc tests revealed that children in the control condition received lower scores than children in the adult protagonist condition for both questions, largest *z* = 3.993, *p* < .001 and also lower scores than in the girl protagonist condition for the first question, *z* = 2.53, *p* = .034, indicating that the questions were mostly easier to answer in the control condition. There was no significant difference for the second question between the control condition and the girl protagonist condition, *z* = 2.19, *p* = .086. There were also no significant differences between both experimental conditions for either of the questions, smallest *z =* 1.75, *p =* .242. As the experimenter provided the correct replies to all participating children, if necessary, we assumed that all children understood the main message from the picture books after engaging with the control questions.

**Table 2:** Duration of distraction in Experiment 1 and Experiment 2.

Condition	*M* (WS1)	*SD* (WS1)	*M* (WS2)	*SD* (WS2)
Adult Protagonist (*n* = 19)	36.58	40.21	53.06	43.08
Girl Protagonist (*n* = 18)	41.87	28.55	50.92	31.73
Control (*n* = 18)	49.83	29.26	41.18	39.34
Dialogic Girl Protagonist (Exp. 2) (*n* = 22)	30.38	18.77	39.64	28.43

Length of distraction in seconds for the estimated marginal means in experiment 1 (WS = waiting situation)

In preliminary analyses for the planned ANOVA, we found almost no significant outliers for any of the conditions. There were two significant outliers in the girl protagonist condition in the first waiting situation, which lay very close to each other. Here, we decided not to exclude them from data analyses, as we cannot state with certainty that they are not within range of normal child behavior due to the modest sample size in each condition.

Data was not normally distributed in some of the situations (e.g., waiting situation 1 in the child protagonist condition), but for others (e.g., waiting situation 2 in the child protagonist condition). Since the repeated measures ANOVA is considered to be robust against violations of normal distribution [e.g., 43], we decided to continue with analyses without transformation. There were no violations of sphericity.

#### Main analyses

Our main interest concerned potential changes in distraction from the first to the second waiting situation as a function of condition. We conducted a 2 (waiting situation: 1, 2) x 3 (condition: adult protagonist, child protagonist, control) repeated measures ANOVA on distraction scores. There was no main effect of waiting situation, *F*(1,63) = 1.14, *p* = .291, *η*_*p*_^*2*^ = .018, no main effect of condition, *F*(1,63) = .24, *p* = .789, *η*_*p*_^*2*^ = .007 and no significant waiting situation by condition interaction effect, *F*(2,63) = 1.23, *p* = .299, *η*_*p*_^*2*^ = .038.

**Fig 6:**
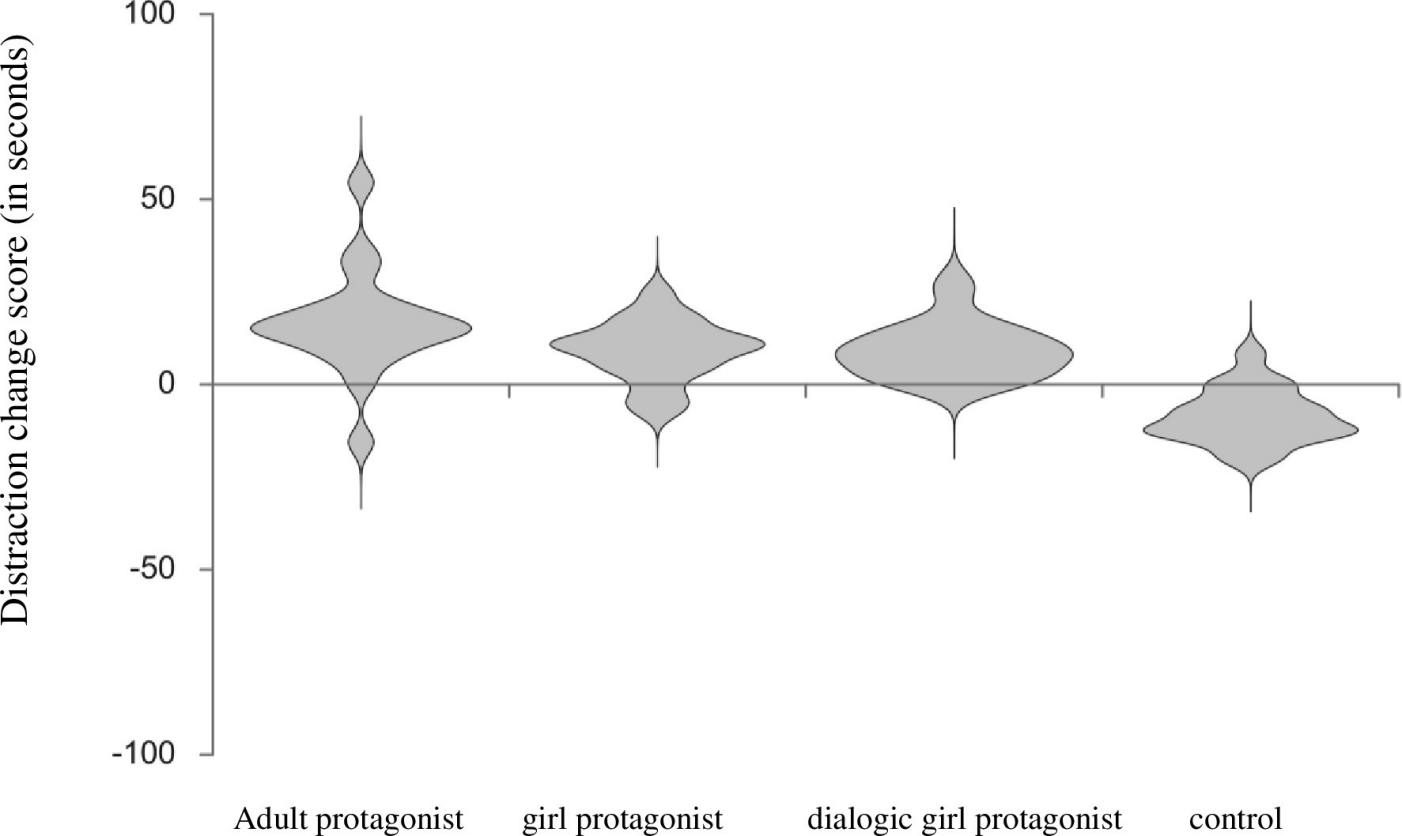
Display of the distraction change score for each condition (Exp.1 & Exp.2).

As a second step, we controlled for previous picture book experience of the participating children, as differences in experience might relate to differences in understanding, encoding and transferring. We therefore conducted a 2 (waiting situation: 1, 2) x 3 (condition: adult protagonist, child protagonist, control) repeated measures ANCOVA on distraction scores, controlling for the items from the picture book experience questionnaire. Data from *n* = 11 children on picture book experience was missing. Regarding the descriptive statistics (see Table 2 for estimated marginal means after controlling for the picture book experience), there was a numerical increase from waiting situation 1 to waiting situation 2 in both waiting situations and a numerical decrease in the control condition. There was no main effect of waiting situation, *F*(1,48) = .31, *p* = .578, *η*_*p*_^*2*^ = .007, no main effect of condition, *F*(2,48) = .16, *p* = .850, *η*_*p*_^*2*^ = .007 and no significant waiting situation by condition interaction effect, *F*(2,48) = 2.63, *p* = .083, *η*_*p*_^*2*^ = .083. Fig 6 displays the change score of distraction (waiting situation 2 –waiting situation 1), that is a positive score indicates an increase in distraction.

In the present study, we examined the influence of picture book content on children’s subsequent behavior with regard to an emotion regulation strategy, namely distraction. Numerical evidence suggests an increase of distraction in both experimental conditions and a decrease in the control condition from waiting situation 1 to waiting situation 2. But statistical analyses did not show any significant results, though there were medium interaction effect sizes.

The results of the present study extend previous research on joint picture book reading by content and age, suggesting that 3-year-old children might not yet be able to infer emotional knowledge from books and apply this knowledge to related situations. A further possibility is that young children need more support to learn to apply an emotion regulation strategy from picture book readings. Therefore, we decided to manipulate the reading situation from a strict adherence to the text to a dialogic reading style in order to support the young children in their learning in a follow-up experiment (Exp. 2). This approach might have higher ecological validity for young children. In addition, past research has shown that interactive reading is more effective in supporting vocabulary skills in young children than strict adherence to the text [8, 9]. Previous research has also demonstrated that mothers tend to interact with their children while reading and ask questions about the content of picture books [6, 7]. Experiment 2 was therefore designed to extend Experiment 1 by investigating the effectiveness of a possibly more naturalistic strategy that supports children’s learning from picture books.

## Experiment 2

Experiment 2 was designed to sample one additional experimental condition which had not been planned a priori to Experiment 1 but was considered to be valuable for exploring the potential and limitations of a short book reading intervention for early use of emotion regulation strategies. The design and procedure were identical to the girl experimental condition in Experiment 1, except that dialogic, instead of standardized, picture book reading occurred. It was therefore planned to compare the dialogic reading condition to the girl protagonist experimental condition and to the control condition from Experiment 1. As we had just begun data collection for Experiment 1 when we planned Experiment 2, we were not able to base the decision which picture book to use in Experiment 2 on these results. We decided to use the girl protagonist picture book, as we assumed it might be slightly more appealing to the children. We hypothesized that the dialogic reading condition would facilitate increased use of distraction, more so than the girl protagonist condition from Experiment 1.

### Method

#### Participants

An additional *n* = 22 (*n* = 11 boys) 3-year-old children (36 months +/-1 month) and their parents participated in Experiment 2. No child had to be excluded. As we intended to calculate one further 3 (conditions) x 2 (waiting situations) ANOVA, the number of participants was reached as a priori determined (*n* = 22 in each condition). Twenty children participated with their mothers and two with their fathers. Maternal age was *m* = 35.95 years (*SD* = 3.50) and paternal age was *m* = 38.27 years (*SD* = 5.61). Seventy-seven percent of mothers und 55% of fathers had a university degree. Seventy-three percent of both mothers’ and fathers’ native language was German. Half of the participating children had siblings. Data collection took place between 2017 (November) and 2018 (June).

#### Design, questionnaires & materials

The study design, questionnaires and materials were the same as in Experiment 1. Data collection largely took place in parallel to Experiment 1 but it began later. Participants were then randomly assigned to one of the four conditions (i.e., three for Exp. 1 and one for Exp. 2). In Exp. 2, internal consistencies for the MDBF were *α* = .87 for `Good/Bad Mood`and *α* = .86 for `Calm-agitated`. The picture book was identical to the picture book used in the experimental condition showing the girl protagonist in Experiment 1. In Experiment 2, the reading style was changed compared to Exp. 1, as described below.

#### Set-up and procedure

In the book reading situation, the experimenter interacted with the child according to a different script compared to Experiment 1. There were scripted, standardized interactions on and about almost every page. While reading the book for the first time, the experimenter asked questions about colors, Anna’s feelings and Anna’s actions in the picture book. When reading the picture book for the second time, the experimenter referred to the child’s prior waiting situation in comparison to Anna’s waiting situation. She asked what the child had been waiting for previously, what color the lamp in his or her waiting situation experience had looked like, what the child had done while waiting, what the child would do when he or she needed to wait again, how the child liked it when the lamp turned green. The intention of this script was to help the child transfer the content from the picture book to their situation in the laboratory. After having finished reading the picture book, the experimenter asked the same control questions as in the experimental conditions in Experiment 1. The complete script is available upon request from the first author.

#### Data coding and interrater reliability

Questionnaires, distraction and negative affect were coded in the same manner as in Experiment 1. The second author coded all videos and the first author coded 23% of these videos for interrater reliability. An additional 6 videos (27%) were coded by the same independent raters as in Experiment 1. Interrater reliability for distraction was ICC (one way random) = .95 and for negative affect, it was ICC (one way random) = .77.

### Results

#### Manipulation checks

We conducted a paired t-test to test for an increase of negative affect from baseline to the first waiting situation in the dialogic reading condition. There was an increase in negative affect, *t*(21) = -2.18, *p* = .041, *d* = .46. Regarding the parental ratings of mental state, there were significant differences for the scale Calm-Agitated with *t*(21) = 6.67, *p* < .001, *d* = 1.42 and for the scale Good/Bad Mood, *t*(21) = 4.47, *p* < .001, *d* = .95 with increases in agitation and bad mood.

Regarding the control questions after picture book reading, we compared the answers from the dialogic reading condition (Exp. 2), the girl protagonist condition and the control condition (Exp. 1) with Kruskal-Wallis tests. For Question 1, there was no significant difference, χ^2^ = 4.91, *p* = .086. For Question 2, there was a significant difference, χ^2^ = 6.04, *p* = .049. Dunn-Bonferroni post hoc tests revealed a significant difference between the control condition and the girl protagonist condition (Exp. 1) with lower scores for the control condition indicating that this question was easier to reply to in the control condition, *z* = 2.44, *p* = .044. There were no significant differences between the dialogic girl protagonist condition and the control condition *z* = -1.46, *p* = .432 or the dialogic girl protagonist condition and the girl protagonist condition, *z* = .98, *p* = .978 for the second question.

#### Main analyses

Looking specifically at the significance of interaction processes in the context of picture book reading, we compared the control condition and the girl protagonist condition from Experiment 1 and the dialogic girl protagonist experimental condition from Experiment 2 (Table 3). We conducted a 2 (waiting situation: 1,2) x 3 (condition: girl protagonist, dialogic girl protagonist, control) repeated measures ANOVA on the duration of distraction. There was no main effect of waiting situation, *F*(1,63) = 1.17, *p* = .283, *η*_*p*_^*2*^ = .018. There was moreover no main effect of condition, *F*(2,63) = 1.69. *p* = .193, *η*_*p*_^*2*^ = .051. There was also no significant waiting situation by condition interaction effect, *F*(2,63) = 1.66, *p* = .198, *η*_*p*_^*2*^ = .050.

**Table 3:** Number of correct replies to the control questions in Experiment 1 and Experiment 2.

Control Question	Condition	Correct reply after first question	Correct reply after second question	Correct reply after looking at the picture book	Correct reply stated by us
1	Adult Protagonist	1 (4.5%)	2 (9.1%)	0	19 (86.4%)
	Girl Protagonist	4 (18.2%)	2 (9.1%)	3 (13.6%)	13 (59.1%)
	Control	10 (45.5%)	4 (18.2%)	1 (4.5%)	7 (31.8%)
	Dialogic Girl Protagonist (Exp. 2)	6 (27.3%)	4 (18.2%)	0	12 (54.5%)
2	Adult Protagonist	1 (4.5%)	2 (9.1%)	2 (9.1%)	17 (77.3%)
	Girl Protagonist	3 (13.6%)	1 (4.5%)	2 (9.1%)	16 (72.7%)
	Control	10 (45.5%)	3 (13.6%)	0	9 (40.9%)
	Dialogic Girl Protagonist (Exp. 2)	5 (22.7%)	4 (18.2%)	0	13 (59.1%)

Total number of correct replies (percentages in brackets) for each control question over each condition

As in experiment 1, we conducted a 2 (waiting situation: 1,2) x 3 (condition: girl protagonist, dialogic girl protagonist, control) repeated measures ANCOVA with the items from the picture book experience questionnaire as covariates to control for previous picture book experience. There was no main effect of waiting situation, *F*(1,51) = .09, *p* = .771, *η*_*p*_^*2*^ = .002. There was moreover no main effect of condition, *F*(2,51) = 1.55. *p* = .223, *η*_*p*_^*2*^ = .057. However, there was a significant waiting situation by condition interaction effect, *F*(2,51) = 3.56, *p* = .036, *η*_*p*_^*2*^ = .122. Our main interest was to compare the change in distraction across waiting situations between conditions. Hence, rather than using omnibus post-hoc pairwise comparisons for each waiting situation, we specifically probed this assumption by conducting independent t-tests between conditions with the change score of the estimated marginals means. There was a significant difference between the control condition and the girl protagonist condition (exp.1), *t*(34) = 6.79, *p* <. 001, *d* = 2.26 and between the control condition and the dialogic girl protagonist condition *t*(38) = -7.60, *p* < .001, *d* = -2.42, but no significant differences between both experimental conditions *t*(38) = -.08, *p* = .936, *d* = -.03. Hence, distraction increased in both experimental conditions compared to the control condition. Regarding within-condition changes, paired t-tests were significant in all three conditions, with increases in both experimental conditions and a decrease in the control condition: girl protagonist condition (exp. 1): *t*(17) = -4.65, *p* < .001, *d* = -1.10, dialogic protagonist condition: *t*(21) = -5.83, *p* < .001, *d* = -1.24, control condition: *t*(17) = 4.99, *p* < .001, *d* = 1.18. To complement this focused post-hoc analysis with a more conservative approach, we also conducted Sidak post-hoc comparisons. To scrutinize the within-between interaction, Sidak post-hoc comparisons were calculated on the basis of an ANCOVA with the distraction change scores (post-pre) as the dependent variable. These tests were not significant between the dialogic girl protagonist and the girl protagonist conditions, *p* = .995; CI [-21.98; 26.20]; nor between the dialogic girl protagonist and the control conditions, *p* = .075, CI [-1.61; 45.50]; nor between the girl protagonist and the control conditions, *p* = .067, CI [-1.28; 49.40].

As an afterthought, we performed four moderation analyses including the three experimental conditions (adult protagonist, child protagonist, dialogic child protagonist) to test whether child reading experience moderated the relationship between distraction in the first and second waiting situation. Detailed analyses can be found in the S1 File. Only the second item of the questionnaire asking about the time spent reading a picture book on an average day significantly moderated the relationship between distraction in both waiting situations for children whose parents reported more than 30 minutes picture book reading on an average day.

## General discussion

The present study investigated whether 3-year-old children changed their use of the emotion regulation strategy distraction in a challenging situation through picture book reading. Young children who had read a picture book that displayed how the girl protagonist distracted herself with toys while waiting subsequently showed more distraction in a waiting situation than young children who had read a picture book that displayed how the same protagonist helped cooking. However, this effect only emerged when controlling for previous picture book experience and when comparing the girl protagonist condition (exp. 1) with the dialogic reading condition and the control condition (exp. 2), but not in experiment 1. Additionally, Sidak post-hoc pairwise comparisons were not significant in contrast to the independent t-tests. There were no significant differences in the change scores of distraction between both experimental conditions in Experiment 2, that is simple text reading versus dialogic picture book reading. Overall, these findings extend previous research on the role of picture book reading for social-emotional behaviors like truth-telling and altruism in children [13, 15] by assessing a different aspect (i.e., emotion regulation) and studying a younger sample.

Young children in the present sample were—to some degree—able to transfer knowledge as complex as an emotion regulation strategy from a picture book to their own situation, suggesting picture book reading as a potential method to help children develop adaptive emotion regulation strategies. A more parsimonious explanation of the present results is that children simply learned a behavioral rule regarding what to do during a wait, without having an insight in the underlying mechanism (i.e., distraction as regulation). Future research could investigate further by providing one group of children with a rationale i.e., distraction in order to feel better, whereas children in the second group might only receive information about rules i.e., “here, we play with toys when we have to wait”. Even if future studies show that 3-year-olds have limited or no explicit understanding of why they are using more distraction after observational learning, the present findings are relevant from a practical, clinical, and theoretical perspective. That is, if children apply more distraction without understanding the rationale, they might have different experiences, such as feeling less negative affect themselves. They might also receive more positive, or at least less negative feedback from their caregivers in waiting situations and thereby implicitly learn to use distraction as an emotion regulation strategy.

Waiting is an everyday activity for 3-year-old children and picture books addressing this aspect of life might help them and their parents to find ideas how to behave adaptively in these situations. In clinical contexts, intervention studies have shown that specifically designed picture books can reduce venipuncture distress in preschool-aged children [44] and reduce preoperative anxiety in children [45]. Together, these findings indicate that young children have an emerging ability to transfer complex knowledge from picture books to their own lives, with the potential to support their development and mental well-being.

Interestingly, there were no significant behavioral changes when we tested the effects of the age of the protagonist (child vs. adult) against the control condition (Exp. 1). There may be at least two potential statistical reasons. First, the standard deviation of the adult protagonist condition was quite large–about 30–40% larger than the standard deviations in the other three conditions. We have no sound explanation for this, especially considering that study procedures were exactly the same for all participating children up to reading the picture book. Regarding the standard deviations in the second waiting situation, it may be that children differed in their learning from an adult model more than they differed in learning from peers. A second potential statistical reason may be the slightly different sample sizes. We had to exclude three children from the adult protagonist condition for the ANCOVA, as their parents had not filled out the picture book experience questionnaire. As there was a trend towards significance and a medium sized interaction effect, it may be that three more children would have changed the significance level. More content-related reasons may be that understanding the usage of an emotion regulation strategy from picture book reading, transferring it to one’s own situation and applying it while being slightly frustrated might be a very difficult undertaking for three-year-olds. Simple text reading might not have supported the children enough to change their behavior.

The present study therefore also focused on possible effects of different reading styles. Unexpectedly, there were no larger increases in distraction when the picture book was read with accompanying dialog rather than purely the text. Nevertheless, one of the control questions posed after reading the picture book was significantly easier to answer in the control condition compared to the girl protagonist condition (Exp. 1), but not compared to the dialogic reading condition (Exp.2). This suggests that the dialogic reading style might facilitate understanding, but not the application of the learned content to the children’s own situation. Further studies could eschew providing children with the correct answers to the control questions, and thereby stating the content of the picture book, as differences in the books’ effectiveness might become more evident in this scenario. Furthermore, in the present study, the experimenter read the picture book with the children. This procedure was different to some studies in which the mother read the picture book [6, 8]. In an imitation study with 18-month-olds, infants’ reproduction of target actions was enhanced when a televised model’s demonstrations were accompanied by a narration that had been developed based on mothers’ naturalistic descriptions of the event [46]. However, a recent study found no significant correlations between parental reading behavior and child word learning or child learning of the moral lessons as portrayed in the picture book [14]. These results are in line with the results of the present study in suggesting that a dialogic reading style might not always facilitate learning from picture books, but that children also benefit from more scripted reading sessions. These results might be particularly relevant for kindergarten or classroom situations where the caregiver or teacher reads the picture book for an entire class, precluding individualized interactions around its content.

Picture books are only one type of media that children enjoy and spend time with, and which also has the potential to teach. The use of digital media has become increasingly common in families with even very young children [47]. Children can learn and benefit from digital media use under certain circumstances, such as during joint media engagement [48, 49]. It is therefore possible that children are able to learn complex strategies, such as an emotion regulation strategy through digital media. Future research could investigate possible methods and identify helping and hindering factors, such as length, potential number of repetitions or dialogic features.

Some limitations of the present study need to be considered. First, the children read a picture book which had been staged in the exact laboratory depicting the exact situation they were experiencing. In everyday life, the settings in children’s books are typically quite different from the child’s current situation. Therefore, the ecological validity of the present procedure was limited. Nevertheless, a recent study with 6- to 8-year-old children showed that these children did not reevaluate their fairness principles after having read a picture book that required generalization to their situation, but after having read a picture book that matched their laboratory task [50]. To test whether even younger children learn to apply an emotion regulation strategy from shared picture book reading in principle, a picture book which was matched to the situation at hand was needed. Moreover, the picture book in the control condition differed significantly from the picture books in the experimental conditions not only by displaying distraction but also by including negative emotions. In future studies, a closer match between books could be achieved by describing an emotion-eliciting event in the book used in control conditions.

A further point regarding construct validity concerns the instrumentalization of distraction. In studies with older children or adults, distraction can be operationalized as a mind-occupying task such as counting backwards [51]. Distraction in studies with younger samples is usually operationalized through toy exploration, as was done in the present study [31, 32, 34]. Toy exploration, however, might also elicit positive emotions and not only reduce negative emotions. Nevertheless, even if the modelled distraction in the present study exceeded the goal of reducing negative affect by also promoting positive affect, children in the experimental conditions were able to observe and apply this regulating behavior from shared picture book reading. These changes would suggest shared picture book reading as a method for teaching emotion regulation.

Furthermore, in experiment 2, the control condition was not read in a dialogic manner. Whilst there is so far no evidence for this assumption, it is possible that different reading styles impact the mood of the children and thereby change their subsequent behavior in a waiting situation. Future studies could therefore include a second control condition which is also read in a dialogic manner.

One additional important factor to consider is that analyses were only significant when we controlled for children’s previous picture book experience. Repetition has been found to decrease the transfer deficit and likely improves encoding variability [e.g., 52, 53]. A recent meta-analysis about the effect of training in joined picture book readings on language development found that the impact was moderated by intervention dosage, such as a lower dosage was associated with minimal impact. They also found a large effect of caregiver book-sharing competence, that is sensitivity during reading [54]. These studies looked at the direct impact of repeated exposure to media. The next logical step would be to assume that a general and not only specific ability to understand and learn from picture books can be fostered through experience. These ideas seem to be underlined by the results of the moderation analyses in the present study, indicating that these children who read picture books for more than 30 minutes on an average day, increased their usage of distraction from the first to the second waiting situation after having read a picture book about applying distraction. It therefore seems that picture book experience is essential in what and how much children are able to take from picture books and should be considered in future research and in promoting content through picture book readings.

A further limitation of the present study is the homogeneity of the sample. As is often the case in developmental research [55], most participants came from a middle-class or upper-middle class background and the majority reported their children owning more than 20 picture books. Children from a lower socio-economic background might comprehend and absorb less information from a picture book for example, if they have access to fewer books at home. A related limitation and a relevant point might also be the young age of the children in the present study. The literature concerned with learning complex patterns through picture book reading has been conducted with older children, namely 3–7 years old [13], and 4–6 years old [15] participants. It may be that the 3-year-old children in the present study were on the verge of learning this transfer. In a previous study, 4-year-old children learned, generalized, and explained facts from a book, whereas 3-year-old children were not able to transfer that knowledge [3]. Future research should therefore extend their focus on different types of picture books, diverse ethnic and sociodemographic backgrounds and different age groups.

In conclusion, the present study tested the potential to use picture book reading to support young children in their use of adaptive emotion regulation strategies. Three-year-old children increased their use of distraction in a frustrating situation as a result of reading a picture book that described how a peer protagonist showed this behavior, but not with an adult model. Future research could investigate age spans that might especially profit from observational learning from media and investigate picture book reading as a teaching or possible intervention method in young children in order to promote their social-emotional development.

## Supporting information

S1 File Additional analyses. (DOCX)
