# Supplementary material for: The effect of picture book reading on young children’s use of an emotion regulation strategy
Source: PLoS One. 2023 Aug 2;18(8):e0289403. doi: 10.1371/journal.pone.0289403 (PMC10395841; doi:10.1371/journal.pone.0289403)
Supplement: S1 File — (DOCX) [file pone.0289403.s001.docx]

**Supporting Information: Additional analyses**

**Possible changes in negative affect**

In order to test whether the duration of negative affect changed between conditions and situations, we performed a 2 (situation: waiting situation 1; waiting situation 2) x 3 (condition: adult protagonist, child protagonist, control) repeated measures ANOVA on the duration of negative affect. There was no significant main effect of situation, *F*(1,63) = 1.04, *p* = .311, *η_p_^2^* = .016. There was no main effect of condition, *F*(2,63) = .10, *p* = .904, *η_p_^2^* = 003*.* Moreover, there was no interaction effect*, F(2,63)* = .41*, p* = .668*, η_p_^2^*= .013*.*

For experiment 2, we also calculated a 2 (situation: waiting situation 1; waiting situation 2) x 3 (condition: dialogic girl protagonist, standardized girl protagonist, control) repeated measures ANOVA on the duration of negative affect. There was no significant main effect of situation, *F*(1,63) = .37, *p* = .544, *η_p_^2^* = .006. There was no main effect of condition, *F*(2,63) = .89, *p* = .418, *η_p_^2^* = 027*.* Moreover, there was no interaction effect*, F(2,63)* = .15*, p* = .859*, η_p_^2^*= .005*.*

**Moderation Analyses**

We calculated moderation analyses including the three experimental conditions (adult protagonist, child protagonist, dialogic child protagonist) to test whether the reading experience moderated the relationship between distraction in the first and second waiting situation. We performed four moderation analyses, one for each item of the reading experience questionnaire using Process macro v.3.5 by Andrew Hayes, which uses ordinary least squares regression, yielding unstandardized coefficients for all effects. Bootstrapping with 5000 samples together with heteroscedasticity consistent standard errors (HC3; Davidson & MacKinnon, 1993) and mean centering were used.

First item: How many picture books does your child own?

The overall model was significant *F*(3,55) = 5.15, *p* = .003, *R^2^* = 18.13 %. The first item of the reading experience questionnaire did not moderate the effect between distraction in both waiting situations, *ΔR^2^* = 6.50, *F*(1,55) = 2.63, *p* = .111, 95% *CI* [-.16, 1.56].

Second item: How much time does your child spend reading a picture book on an average day?

The overall model was significant *F*(3,55) = 10.98, *p* < .001, *R^2^* = 25.32 %. The second item of the reading experience questionnaire moderated the effect between distraction in both waiting situations significantly, *ΔR^2^* = 13.57, *F*(1,55) = 8.64, *p* = .005, 95% *CI* [.26; 1.18]. The moderator was significant for children whose parents reported more than 30 minutes picture book reading on an average day.


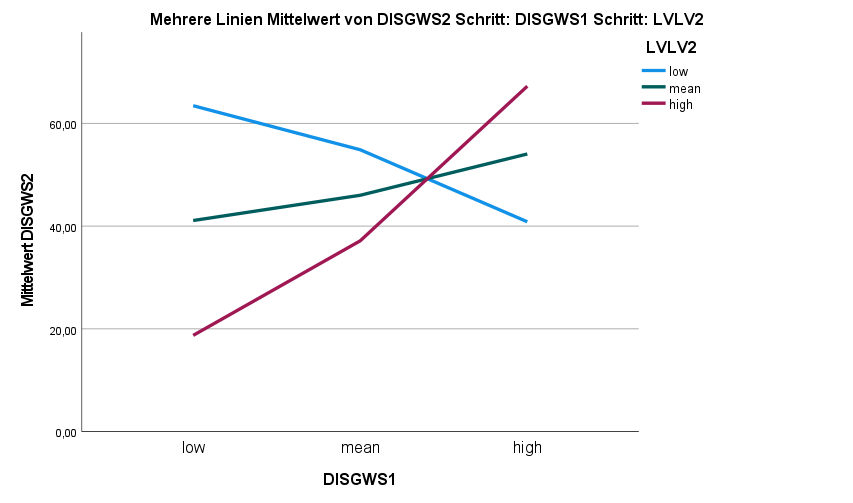


Distraction in Waiting Situation 2

Distraction in Waiting Situation 1

*S1_Figure.* The interaction effect of the second item of the picture book experience questionnaire and use of distraction in the first waiting situation on use of distraction in the second waiting situation.

Third item: How well does your child understand a new picture book?

The overall model was not significant *F*(3,55) = 1.69, *p* = .181, *R^2^* = 12.11 %. The interaction effect was also not significant, *ΔR^2^* = 1.12, *F*(1,55) = .36, *p* = .549, 95% *CI* [-.46, .68].

Fourth item: How much does your child like to read picture books?

The overall model was not significant *F*(3,55) = 1.35, *p* = .267, *R^2^* = 11.81 %. The interaction effect was also not significant, *ΔR^2^* = 0.52% , *F*(1,55) = .17, *p* = .681, 95% *CI* [-.60, 1.29].
